# The Emerging Role of Myeloid-Derived Suppressor Cells in the Glioma Immune Suppressive Microenvironment

**DOI:** 10.3389/fimmu.2020.00737

**Published:** 2020-04-24

**Authors:** Yajing Mi, Na Guo, Jing Luan, Jianghong Cheng, Zhifang Hu, Pengtao Jiang, Weilin Jin, Xingchun Gao

**Affiliations:** ^1^Shaanxi Key Laboratory of Brain Disorders, Institute of Basic Medical Sciences, Xi’an Medical University, Xi’an, China; ^2^Key Lab for Thin Film and Microfabrication Technology, Department of Instrument Science and Engineering, School of Electronic Information and Electronic Engineering, Institute of Nano Biomedicine and Engineering, Ministry of Education, Shanghai Jiao Tong University, Shanghai, China

**Keywords:** MDSC, glioma, immunosuppression, tumor microenvironment, therapeutic target

## Abstract

Myeloid-derived suppressor cells (MDSCs) are a heterogeneous group of myeloid progenitor and precursor cells at different stages of differentiation, which play an important role in tumor immunosuppression. Glioma is the most common and deadliest primary malignant tumor of the brain, and ample evidence supports key contributions of MDSCs to the immunosuppressive tumor microenvironment, which is a key factor stimulating glioma progression. In this review, we summarize the source and characterization of MDSCs, discuss their immunosuppressive functions, and current approaches that target MDSCs for tumor control. Overall, the review provides insights into the roles of MDSC immunosuppression in the glioma microenvironment and suggests that MDSC control is a powerful cellular therapeutic target for currently incurable glioma tumors.

## Introduction

Glioma is the most common primary malignant tumor of the brain, and is characterized by high proliferation rates, and migration and invasion abilities. Comprehensive treatment includes surgical resection combined with radiotherapy and chemotherapy; however, such approaches are generally ineffective. The median survival time of patients with the most malignant glioma is approximately 1–2 years despite aggressive therapy, including surgery, radiotherapy and chemotherapy (1). Hence, development of more effective treatments is urgently required. Recently, immunotherapeutic approaches have been developed for cancer therapy, with exciting progress for some cancers; however, there are specific challenges for glioma immunotherapy (2). Many factors may contribute to these difficulties, including the blood-brain barrier (BBB), antigenic and genetic heterogeneity, and the tumor microenvironment (TME).

## The Immune Microenvironment in Glioma

The TME is the dynamic milieu of a tumor, including the extracellular matrix (ECM), signaling molecules, stromal cells, and immune cells, which influence the growth and evolution of tumor cells. The glioma microenvironment differs from other solid tumors, because gliomas are located in the brain, which is an immune privileged organ, protected by the BBB, where cells of the peripheral immune system are prevented from entering under normal conditions. However, inflammation and tumor growth can disrupt the BBB (3, 4). The strong immunosuppressive TME of gliomas has led them to be referred to in the literature as “cold tumors” (5). Many studies have demonstrated that cytokines, chemokines, and regulatory immune-suppressive cells (6, 7), such as TGF-β, IL-10, prostaglandin E2, NKT cells, T/B regulatory cells (T/Breg), tumor-associated macrophages/microglia (TAMs), and myeloid-derived suppressor cells (MDSCs) (8), create a specific immunosuppressive TME, which is important for anti-tumor responses and glioma progression. All these proved that MDSCs are powerful inhibitors of anti-tumor immune responses in glioma, hence targeting MDSCs will be beneficial for patients with these tumors. Here, we review the phenotypic characteristics of MDSCs, as well as their mechanisms of development and activation, and strategies for MDSC-depletion.

## Source and Characterization of MDSCS

Myeloid-derived suppressor cells are a heterogeneous group of myeloid progenitor and precursor cells, comprising macrophages, granulocytes, and dendritic cells (DCs) at different stages of differentiation. In healthy individuals, immature myeloid cells (IMCs) quickly differentiate into mature macrophages, granulocytes, or DCs (9); however, under pathological conditions, such as in patients with cancer, chronic inflammatory conditions prevent IMC differentiation into mature myeloid cells, resulting in MDSC accumulation (10). In cancer patients, MDSCs are defined as cells that co-express the myeloid differentiation markers, CD11b and CD33, while lacking markers of mature lymphoid and myeloid cells, such as the MHC class II molecule, HLA-DR (11). There are three main types of MDSCs: granulocytic or polymorphic nuclear MDSCs (G/PMN-MDSCs), mononuclear MDSCs (M-MDSC), and early-stage MDSCs (eMDSCs). In humans, M-MDSCs are characterized as CD11b^+^CD14^+^CD33^+^HLA-DR^*low*/−^CD15^–^, PMN-MDSCs as CD11b^+^CD14^–^CD33^+^HLA-DR^*low*/−^CD15^+^ (or CD66^+^), and eMDSCs as Lin^–^ (i.e., CD3^–^, CD14^–^, CD15^–^, CD19^–^, CD56^–^, HLA-DR^–^, and CD33^+^. In mouse, MDSCs are defined as cells that co-express CD11b and Gr-1 (12), where Gr-1 is a cell surface antigen, and can be divided into Ly6C and Ly6G. Hence, M-MDSCs are characterized as CD11b^+^, Ly6C^+^, Ly6G^–^, according to their relative expression levels of Ly6G and Ly6C, whereas PMN-MDSCs are CD11b^+^, Ly6C^–^, Ly6G^+^, and eMDSCs have yet to be defined.

In humans, M-MDSCs can be distinguished from monocytes base on the expression of the MHC class II molecule HLA-DR (13). The PMN-MDSCs and neutrophils can be separated on the base of their low-density properties when using a standard Ficoll gradient. Recently, Condamine T. et al. identified that lectin-type oxidized low-density lipoprotein receptor-1 (LOX-1) as a potential marker of human PMN-MDSC (12). Moreover, the M-MDSCs and PMN-MDSCs also display specific cell-death-associated program. Haverkamp et al. demonstrated that the anti-apoptotic molecules c-FLIP [cellular FLICE (FADD-like IL-1β-converting enzyme)-inhibitory protein] is required for monocytic MDSC development (14). Fiore et al. reported that c-FLIP can activate many transcription of several immunosuppression-related genes in part through nuclear factor kappa-light-chain-enhancer of activated B cells (NFκB) activation, and then plays an important role in re-programming monocytes into MDSCs without affecting cell survival, but not affects the conversion from neutrophils into PMN-MDSCs (15).

As we known, the basic functional characteristic of MDSCs is suppressing immune cells, mainly T-cells and lesser B and NK cells (16). Groth et al. reported that M-MDSCs showed a higher immunosuppressive capacity compared to PMN-MDSC, which directly suppress T cell function or induce the generation of Treg cells by secreting TGF-β and IL-10 (17). Trovato et al. showed that M-MDSCs are the most potent myeloid subset to halt T cell proliferation in patients with pancreatic ductal carcinoma (18). Moreover, plasticity and function of MDSCs are strictly regulated by the activation of specific molecular pathways (13), preferentially driven by signal transducer and activator of transcription 3 (STAT3), and CCAAT/enhancer binding proteinβ (C/EBPβ). Upregulation of STAT3 is a hallmark of MDSCs, which plays a central role in regulating MDSC expansion and tolerogenicity (19). Upregulation of C/EBP-β is also associated with the expansion of MDSCs populations (19). Furthermore, C/EBP-β controls the controls the immunosuppressive activity of MDSC through regulating the expression of arginase (ARG1) and inducible nitric oxide synthase (iNOS) (20, 21).

## Myeloid-Derived Suppressor Cells in Glioma

Glioma is a central nervous system (CNS) tumor, and the immunosuppressive TME of glioma likely depends on depressed T cell function, through accumulation of immunosuppressive leukocytes, such as MDSCs and regulatory T-cells (Tregs). MDSCs number is usually relatively larger in the spleen, blood, and tumors, and correlates with the tumor stage and chemotherapy response (22). In glioma patients, the intratumoral density of MDSCs also increases during glioma progression and correlates with patient survival (23). Gielen et al. reported that MDSCs increased in blood samples from patients with glioma compared with healthy donors. (24) and Raychaudhuri et al. found that patients with glioblastoma multiforme (GBM) had elevated levels of MDSCs in their peripheral blood (25), and the majority of the MDSCs were neutrophilic CD15^+^ CD14^–^ (PMN-MDSC, 82%), followed by lineage-negative (eMDSCs, 15%), and monocytic (M-MDSC, 3%). Gielen et al. showed that MDSCs were significantly increased among peripheral blood mononuclear cells from patients with GBM, but only slightly and non-significantly increased in patients with grade II or III glioma, and MDSCs found in tumor material are almost exclusively CD15-positive (24). Dubinski et al. also revealed that the frequency of CD14^high^CD15^pos^ M-MDSCs and CD14^low^CD15^pos^ PMN-MDSCs was significantly higher in peripheral blood of GBM patients compared with healthy donors (26). These studies indicate the expansion of PMN and M-MDSC subtypes in patients with glioma, and M-MDSCs were the most abundant MDSC subpopulation in the blood, while PMN-MDSCs were dominant in glioma tissue. Further, Raychaudhuri et al. found that MDSCs represented 5.4 ± 1.8% of total cells in human GBM tumors; the majority were lineage negative (CD14^–^CD15^–^) eMDSCs, followed by PMN-MDSC (CD15^+^CD14^–^), and M-MDSC (CD15^–^CD14^+^) subtypes. In murine GBM tumors, MDSCs comprised 8.06 ± 0.78% of total cells, of which more were M-MDSC than G-MDSC (27). Hence, it is established that the types of MDSCs differ in tumor tissues compared with the circulation (blood), while detection of the predominant MDSC type in glioma requires further research, since the TME may impact MDSC migration or differentiation.

## MDSC Expansion and Recruitment

Myeloid-derived suppressor cells are present at relatively low levels under normal conditions, but are recruited and differentiate in various pathologic situations, such as during autoimmune encephalitis, bacterial infections, and tumors. Jennifer et al. reported that the healthy donor human CD14^+^ monocytes could acquire MDSC phenotypes (reduced CD14 but not CD11b expression) and immunosuppressive properties (increased immunosuppressive interleukin-10, transforming growth factor-β (TGF-β), and B7-H1 expression) when cultured with human glioblastoma cell lines (28). Kumar et al. also found that normal human monocytes could become M-MDSCs when cultured in glioma-conditioned media under hypoxic conditions (29).

**FIGURE 1:**
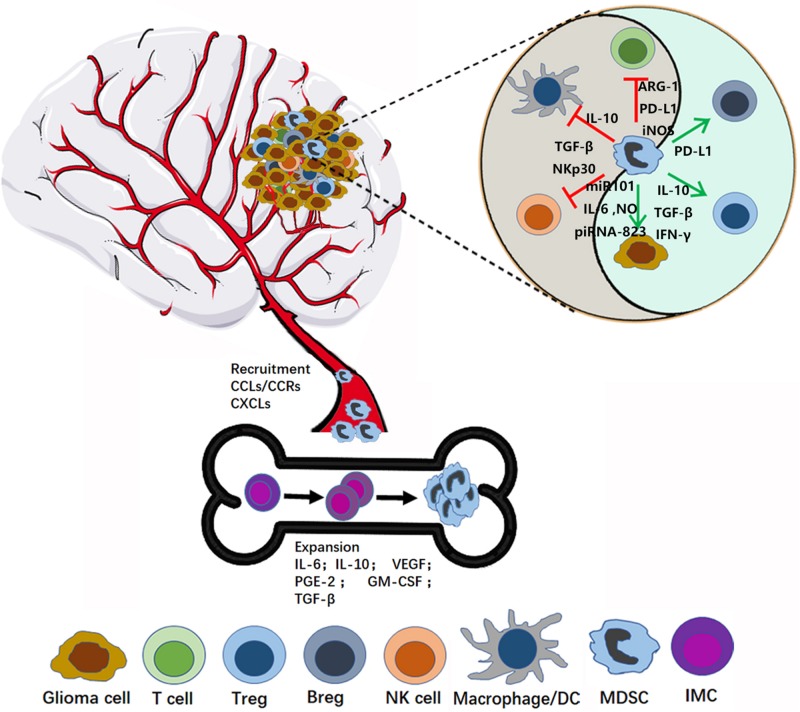
MDSC recruitment and activation in glioma microenvironment. In the bone marrow, MDSCs originate from immature myeloid cells (IMC), and then expand and migrate to the glioma site through the interaction between CCR and respective chemokines (CCL). In the tumor microenvironment, MDSCs play immunosuppression role by inhibiting the anti-tumor activity of cytotoxic T cells, suppressing the NK, Macrophage and Dendritic cells (DCs) function, expansion, and promoting Tregs and Bregs.

A variety of inflammatory mediators can induce MDSC expression and recruitment (Figure 1). Jiang et al. reported that interleukin-6 (IL-6) can promote the amplification and immunosuppressive function of breast cancer MDSCs *in vitro* and *in vivo*, by inducing dysfunction of SOCS3 and activation of signal transducer and transcription activator 3 (STAT3)-signaling (30). Gielen et al. reported that STAT3 also regulates MDSC amplification through expression of S100A8/9 in gliomas (31). Tumor-derived granulocyte macrophage colony-stimulating factor (GM-CSF) has an important role in the expansion of MDSCs, both *in vitro* and *in vivo* (32–34). Importantly, Marigo et al. found that GM-CSF and IL-6 allowed rapid and efficient generation of MDSCs from precursors present in mouse and human bone marrow (35). Moreover, various other tumor-derived factors, such as prostaglandin-E2 (PGE2) (36), IL-10 (37), VEGF (38), and TGF-β (39–41), have been suggested to contribute to the induction and expansion of MDSCs (36), and these factors are also derived from glioma cells. Albulescu et al. showed that IL-6, IL-1β, TNF-α, IL-10, VEGF, FGF-2, IL-8, IL-2, and GM-CSF were upregulated in gliomas (42). Further, many studies have shown that PGE2 is overexpressed in glioma (43). Together, these data suggest that glioma cells can stimulate the expansion of MDSCs by secreting numerous well-studied factors (IL-6, IL-10, VEGF, PGE-2, GM-CSF, and TGF-β2).

Chemokines are a family of 8–14 kDa chemoattractant cytokines secreted by cells, which have important roles in regulating cells trafficking (44). Multiple chemokines are involved in recruiting MDSCs in different cancer models (45–47). Chemokine (C-C motif) ligand (CCL) 2 and its receptors, chemokine (C-C motif) receptor (CCR) 2, 4, and 5, have key roles in attraction of M-MDSCs (48, 49). In particular, microenvironment-derived CCL-2 can recruit MDSCs to cancer sites via CCL2-CCR2 interaction (50). Furthermore, Vakilian et al. reviewed the CCL2/CCR2 signaling pathway in glioma and found that it plays a dual role in mediating early tumor immunosurveillance and sustaining tumor growth and progression (51). IL-8 (CXCL8) is a pro-inflammatory chemokine produced by many cell types, including glioma, and can promote MDSC trafficking into the tumor microenvironment through the IL-8/IL-8R axis (52, 53). CXC chemokine ligand 2 (CXCL2), also referred to as macrophage inflammatory protein-2 (MIP-2), has a pivotal role in recruiting MDSCs to tumor stroma (54). Kammerer et al. found that *CXCL2* was an immune response gene in glioma; however, whether expression of this gene is altered in tumor cells or cells in the TME was not determined (55). Interestingly, Bruyère et al. found that inhibition of CXCL2 expression in Hs683 glioma cells using siRNA markedly impaired cell proliferation (56). Overall, these results suggest that high levels of CXCL2 expression are important for glioma progression; however, the mechanism regulating MDSC recruitment requires clarification.

## MDSC-Induced Immunosuppression in Gliomas

Myeloid-derived suppressor cells indisputably induce immunosuppression and promote tumor development. Numerous mechanisms by which MDSCs inhibit immune responses have been reported, inducing inhibition of the anti-tumor activity of cytotoxic T cells, suppression of NK cell, macrophage, and dendritic cell (DC) function, and induction of Tregs and Bregs. In this section, we summarize the function of MDSCs in glioma development in detail (Figure 1).

### Inhibition of T Cell Function

T cells, particularly cytotoxic T cells, have important roles in tumor-inhibition, and there is substantial evidence that MDSCs can inhibit T cell function via multiple mechanisms. MDSCs are well known to induce oxidative stress by secreting ROS and nitrogen species (RNS). The main pathways of ROS production are related to the NADPH oxidases (NOX) (57), and RNS are produced by the activation of ARG1 or iNOS (NOS2) in different MDSC subsets (58). These reactive species can inhibit T cell growth through interfering with the expression of the CD3ζ chain and induction of apoptosis (59, 60). Moreover, intratumoral RNS production can inhibit the T cell migration by inducing the CCL2 chemokine nitration (61). MDSC can also deplete metabolites and factors which are critical for T cell functions. MDSCs deplete L-arginine which inhibits T cell growth and induce apoptosis from the microenvironment by enhancing the activity of ARG1, inducible iNOS and increase the uptake mediated by the CAT-2B transporter (62, 63). Tryptophan (Trp)-catabolizing enzymes such as Indoleamine 2,3-dioxygenase (IDO) have been shown to be involved in tumor immune escape. Upregulation of IDO1 in MDSC and tumor cells leads to Trp depletion that impairs cytotoxic T cell responses and survival (64–66). HIF1-α is produced in response to hypoxia in the TME and can induce PD-L1 expression on MDSCs. Further, blockade of PD-L1 can inhibit MDSC-mediated T cell suppression, through modulating MDSC cytokine production (67).

### Inhibiting NK Cell Function

NK cells are a critical component of innate immunity and can eradicate gliomas without T cell cooperation (68). Fortin et al. found that the MDSCs can suppress the function of NK cells via reactive oxygen species (ROS) production (69), while Li et al. showed that membrane-bound TGF-β1 on MDSCs can induce NK cell anergy (70). Further, Hoechst et al. reported that MDSCs suppress NK cell cytotoxicity and cytokine release through contact with the NK cell receptor, NKp30 (71). Bruno et al. recently reviewed the interactions of MDSCs and NK cells and determined that cross-talk between these types of cells can impact tumor progression and angiogenesis (72).

### Inhibiting Macrophage and DC Function

Microglia/macrophages are among the most common cells in brain tumors (73), and MDSCs can modulate functions of macrophages (74). Sinha et al. proved that cross-talk between MDSCs and macrophages skews macrophages toward an M2 phenotype by cell-cell contact, and decreasethe production of IL-12 of macrophage (75). The downregulation of IL-12 is further exacerbated by the macrophages themselves, because the production of IL-10 of macrophages is also promoted by MDSCs (76). Interestingly, Pinton et al. found that the bone marrow-derived macrophages (BMDM) exerted a strong immunosuppression in center of the glioma, but brain-resident microglial cells (MG) showed little or no suppression (77). So, the relationship between the MDSC and macrophages need further research in tumor especially glioma. DCs are the most powerful antigen-presenting cells and a DC vaccine has potential to improve clinical outcomes of patients with glioma (78, 79). Hu et al. proved that MDSCs can inhibit IL-12 production and suppress T cell stimulation of DCs through IL-10 production (80). These results suggest that MDSCs can modulate the functions of macrophages and DCs through different mechanisms.

### Treg Expansion and Differentiation

Tregs are suppressor T cells, which can inhibit the induction and proliferation of cytokine-secreting effector T cells (81). Induction of Treg activity is important for the evasion of immunosurveillance by malignant gliomas and correlates with glioma progression (82–84). Huang et al. found that MDSCs can induce Treg generation, dependent on soluble factors, such as IL-10, TGF-β, and IFN-γ (85, 86). In addition, Hoechst et al. showed that MDCSs can catalyze the trans-differentiation of Th17 cells into Tregs, dependent on MDSC-derived TGF-β and retinoic acid (87). These data demonstrate that MDSCs can regulate Treg expansion and induce Th17 cells to differentiate into Tregs.

### Promotion of Immunosuppressive B Cells

Regulatory B cells (Bregs) are a population of immunosuppressive cells that support immunological tolerance (88). Recently, glioma-infiltrating B cells were shown to have strong immune suppressor functions in suppressing CD8^+^ T cells and inducing Tregs (89). Lee-Chang et al. found that MDSCs produced numerous microvesicles (MV) containing PD-L1. Bregs can take up these MVs via receptor-mediated endocytosis, followed by endocytic recycling toward the plasma membrane. These results indicate that MDSCs can promote Breg-mediated immunosuppressive functions, at least in part via transfer of membrane-bound PD-L1.

Besides, MDSC can also directly support tumor (or cancer stem cells) growth through impacting on angiogenesis, invasion and metastasis, and promote cancer cell stemeness (74). MDSCs can produce angiogenic factors such as VEGF and basic fibroblast growth factor (bFGF) to promote tumor angiogenesis (90, 91). Moreover, Li et al. even found that MDSCs directly incorporated into tumor endothelium through acquiring endothelial cell (EC) properties (91). Furthermore, MDSCs promote tumor invasion and metastasis by two mechanisms: (i) increasing the production of multiple matrix metalloproteinases (MMPs), which play an important role in matrix degradation, and chemokines to create a pre-metastatic environment, and (ii) fusing with tumor cells to promote the metastatic process (92, 93). Several studies showed that MDSCs promote cancer cell stemness through inducing miRNA (94), production of IL-6 and NO (95), inducing piRNA-823 expression and DNMT3B activation (96) (Figure 1).

## Targeting MDSCS in Glioma Therapy

**FIGURE 2:**
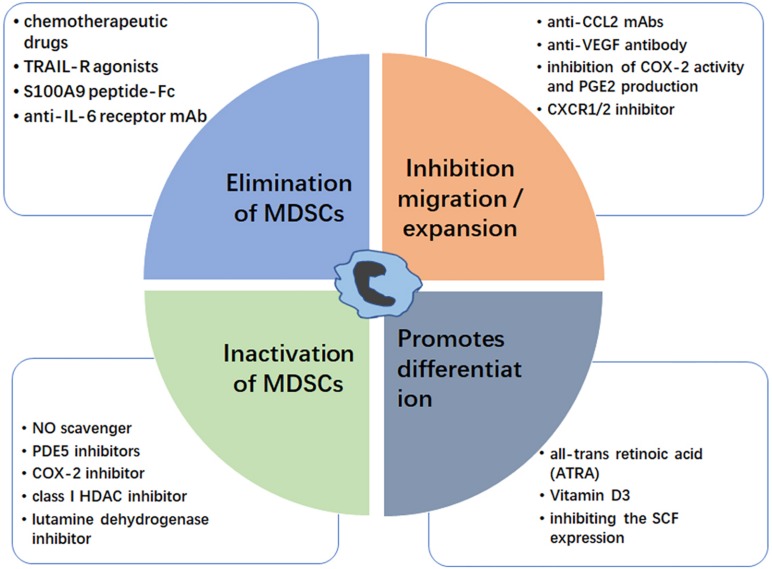
Strategies for targeting MDSC. The MDSC modulation could be achieved by elimination of MDSCs (blue box), inhibition of MDSC migration/expansion (orange box), inactivation of MDSCs (green box), and promoting MDSC differentiation (gray box). Examples for each therapeutic approach are shown.

Myeloid-derived suppressor cells are established as a central immunosuppressive factor, which promotes tumor progression. Therefore, control of the number and/or function of MDSCs would be a powerful anti-tumor therapy. Below, we summarize current targeting approaches for MDSC control (Figure 2).

### Elimination of MDSCs

Elimination of MDSCs inhibits tumor progression by enhancing antitumor responses, and there are many reports that chemotherapy drugs can eliminate MDSCs. Suzuki et al. first proved that the chemotherapeutic drug, gemcitabine, can specifically reduce MDSCs, with no significant reductions in other immune cells, such as T cells, B cells, macrophages, or NK cells, in tumor-bearing animals (97). This immunomodulating capacity of gemcitabine could be useful to treat glioma (98). Otvos et al. showed that gemcitabine and 5-FU were selectively cytotoxic to MDSCs, with 5-FU showing greater efficacy in depleting MDSCs and inducing MDSC apoptosis *in vivo* and *in vitro* (99). Ugel et al. proved that gemcitabine and 5-FU mainly target M-MDSCs (100). Furthermore, low dose 5-FU can selectively deplete MDSCs, resulting in prolonged survival in a glioma mouse model (101). Recently, David et al. found that the orally bioavailable 5-FU prodrug, capecitabine in combination with bevacizumab can reduce the circulating levels of MDSC in GBM patients (102). Many studies support a role for NF-related apoptosis-induced ligand receptors (TRAIL-Rs) act as potential targets for selective elimination of MDSCs (103). Dominguez et al. proved that DS-8273a, an agonistic TRAIL-R2 (DR5) antibody, could eliminate MDSCs without affecting lymphoid or mature myeloid cells (104), and Nagane et al. found that anti-DR5 mAb treatments significantly suppressed growth of subcutaneous glioma xenografts until complete regression (105). Murat et al. found that glioma patients with high expression of S100A8 and S100A9 are related with short survival (106). Recently, Qin et al. proved that S100A9 peptide-Fc fusion (pepti-body) reagents can deplete blood and splenic MDSCs in mouse tumor models (107, 108). Further, many researches proved that anti IL-6 therapy showed potential benefits for treating various human cancers including glioma (109, 110), Sumida et al. found that an anti-IL-6 receptor monoclonal antibody (mAb) could eliminate MDSCs and inhibit tumor growth by enhancing T-cell responses, and that its therapeutic effect was enhanced by combination with gemcitabine (111). Together, these data suggest that MDSCs can be directly depleted using various agents, including chemotherapy drugs, peptides, and mAbs; however, the mechanisms underlying MDSC elimination require further elucidation.

### Inhibition of MDSC Migration and Expansion

Inhibiting MDSC migration and expansion is another strategy for tumor therapy. Glioma cells are known to secrete factors important for MDSC migration and expansion. Zhu et al. proved that systemic administration of neutralizing anti-CCL2 mAbs can block recruitment and decrease the number of MDSCs in the TME, providing significant survival benefits in mouse GL261 glioma and human U87 glioma xenograft models (112). Chen et al. reported that anti-VEGF antibody can effectively decrease the recruitment of MDSCs to tumor tissue (113), while anti-VEGF treatment has been proposed to enhance the survival and quality of life in glioma patients (114, 115). Many studies proved the protective effect of COX-2 inhibitors on glioma (116, 117), and the use of non-steroidal anti-inflammatory drugs was significantly associated with a lower risk of glioma (118, 119). Obermajer et al. demonstrated that inhibition of cyclooxygenase (COX-2) activity and PGE2 production can reduce the accumulation of human MDSCs in the ovarian cancer environment (45, 120). Sinha et al. proved that the COX-2 inhibitor, SC58236, delays primary tumor growth and reduces MDSC accumulation in spontaneously metastatic BALB/c-derived 4T1 mammary carcinoma mouse (121). The chemokine receptor CXCR2 is a receptor of CXCL2 which was highly expressed in PMN-MDSC, andplay an important role in MDSCs recruitment (122). SX-682, a CXCR1/2 inhibitor, could block tumor MDSC recruitment and enhance T cell activation and anti-tumor immunity through various forms of immunotherapy (123, 124).

### Inactivation of MDSCs

Once MDSCs migrate into the TME, they suppress anti-tumor responses via various mechanisms, hence impairment of MDSC activity is another strategy to target these cells. Increased production of NO and ROS facilitates MDSCs to suppress CD8^+^ T cell responses. The ROS scavenger, N-acetylcysteine (NAC), can inhibit MDSC function (108), while the NO scavenger, carboxy-PTIO (C-PTIO), can reduce the immunosuppressive activity of MDSCs and restore impaired CTL function by inhibiting the NO production (125). Activation of iNOS and ARG-1 also has key roles in MDSC activation. Phosphodiesterase-5 (PDE5) inhibitors includesildenafil, tadalafil, and vardenafil, they are emerged as a promising approach to inhibit proliferation, motility and invasion of certain cancer cells including glioma (126). These inhibitors are reported to suppress both iNOS and ARG-1 activities in MDSC, thereby decrease MDSC immunosuppressive functions (127, 128). Moreover, COX-2 is required for the production of PGE2 (129) and correlates positively with ARG-1 expression in MDSCs (130); hence, COX-2 inhibitors, such as celecoxib or acetylsalicylic acid, can suppress gliomagenesis by MDSC activation (131, 132). Entinostat (MS-275), a class I HDAC inhibitor in pre-clinical testing for glioblastoma, can inhibit MDSC function and exhibit antitumor effect in murine models of lung and renal cell carcinoma (133, 134).

It has been reported that the glutaminolysis contributes to MDSC function (135), this means glutamate metabolism (maintaining optimal glutamine or glutamate levels) is critical in MDSC-mediated immunosuppression phenomenon. As we know, glutamate is one of the main excitatory neurotransmitters in the central nervous system (CNS) (136). And in glioma microenvironments, the glutamate concentration is 400 times that of normal brain tissue (137). This high glutamate concentration is benefit for glioma cell growth and affects MDSCs function, so nhibition of glutamine metabolism is suggested as an attractive and druggable therapeutic target especially in glioma (138). The glutamine dehydrogenase inhibitor, epigallocatechin gallate (EGCG), which has been proved to induce apoptosis and inhibit proliferation of glioma cell (139), also could reverse MDSC activity (140).

### Promotion of MDSC Differentiation

Promotion of MDSC differentiation into mature myeloid cells is a simple approach to inhibition of the immune-suppressive functions of MDSCs. All-trans retinoic acid (ATRA), a natural oxidative metabolite of vitamin A, can promote differentiation of MDSCs into mature myeloid cells (141). Mirza et al. reported that patients with cancer treated with ATRA exhibit improved myeloid/lymphoid DC ratios and immune responses (142). Further, Nefedova et al. found that the ATRA can active ERK1/2 MAPK signaling pathway, which increases the production of glutathione and reduces ROS levels, thus promoting MDSC differentiation (143). Recently, wang et al. proved that ATRA can induce asymmetric division of GSCs from the U87MG cell line, suggesting a therapeutic potential for glioma (144, 145). Similar to ATRA, vitamin D3 can induce immature MDSCs to be differentiated into dendritic and macrophages cells, and then displays anti-proliferative effects in a wide variety of cancer types including glioblastoma multiforme (GBM), (146–148). Moreover, Pan et al. proved that stem-cell factor (SCF), which is secreted by various cancers, including glioma (149), can decrease MDSC differentiation, resulting in MDSC expansion, while inhibiting SCF expression using siRNA can reduce MDSC accumulation (150).

## Conclusion and Future Prospects

To date, immunotherapeutic strategies have proven to be effective against various tumors, and researchers are increasingly focusing on immunotherapy for patients with glioma. Although significant progress has been made, some challenges must be overcome (7). There is substantial evidence that MDSCs are important immunosuppressors (11), hence targeting MDSC immune suppressive features has potential as an anti-tumor therapy approach in glioma (151); however, the mechanisms underlying MDSC activity in glioma require further elucidation. In recent years, several strategies have been investigated, such as elimination of MDSCs, inhibition of MDSC migration and expansion, inactivation of MDSCs, and promotion of MDSC differentiation. In summary, control of MDSCs is a powerful cellular therapeutic target for patients with glioma; nevertheless, further basic and clinical research is required in this field.

## Author Contributions

YM, NG, and JL conceived and structured the manuscript. JC, ZH, and PJ drafted the manuscript and figure. XG evaluated and reviewed the manuscript structure, ideas, and science. WJ conceived the topic and revised the manuscript. All authors read and approved the final manuscript.

## Conflict of Interest

The authors declare that the research was conducted in the absence of any commercial or financial relationships that could be construed as a potential conflict of interest.
